# Serum MiRNA as Predictive and Prognosis Biomarker in Advanced Stage Non-small Cell Lung Cancer in Indonesia

**DOI:** 10.3779/j.issn.1009-3419.2020.104.02

**Published:** 2020-05-20

**Authors:** Arif R HANAFI, Achmad M JAYUSMAN, Serafim ALFASUNU, Ahmad H SADEWA, Dibyo PRAMONO, Didik S HERIYANTO, Sofia M HARYANA

**Affiliations:** 1 Department of Pulmonology, "Dharmais" Hospital National Cancer Center, Jakarta 11420, Indonesia; 2 Faculty of Medicine, Gadjah Mada University, Yogyakarta 55281, Indonesia

**Keywords:** Advanced stage NSCLC, Serum miRNA, Survival

## Abstract

**Background and objective:**

Lung cancer is the most common cause of death in men in the world and in Indonesia where non-small cell carcinoma lung cancer (NSCLC) constitutes 85% of all lung cancer cases. The high mortality rate is due to a poor prognosis and is often diagnosed as having advanced stages. If it is known at the initial stage, the prognosis of lung cancer will be better. Prognosis can be predicted with a marker of prognostic biology, one of which is micro RNA (miRNA). This study aims to prove that serum miRNA can be predictive biological marker and prognosis in NSCLC patients in Indonesia.

**Methods:**

This study was cohort retrospective among 52 subjects in "Dharmais" Hospital National Cancer Center. Sample was obtained from patients' serum. MiR-34, miR-148, miR-155 and miR-222 serum are measured through Real-Time PCR (qPCR). Data were analyzed and interpreted with descriptive analysis, bivariate analysis (*Mann Whitney-U* for two type of variables or *Kruskal-Wallis* for more than two type of variables. *Kaplan-Meier* analysis was used to know association between characteristic which are sociodemographic, performance status, clinico-pathology, and survival rate in miRNA expression.

**Results:**

From this study, miRNA expression: miR-34 (46.15%), miR-148 (23.08%), miR-155 (40.38%) and miR-222 (32.69%). Performance status score was statistically significant correlation with miR-148 (*P*=0.049) and miR-222 (*P*=0.018). High miR-34 is associated with multiple M1b metastatic type (*P*=0.020), cancer cell type (adenocarcinoma, *P*=0.009) and adenocarcinoma epidermal growth factor receptor (*EGFR*) mutation (negative, *P*=0.031). There was a significant correlation between the high miR-222 as a poor prognosis in advanced stage NSCLC with M1b metastasis (Median Survival/MS: 27 d, *P*=0.049) and positive *EGFR* mutations (MS: 74 d, *P*=0.049) and correlation of miR-155 with adenocarcinoma (MS: 69 d, *P*=0.034) and positive *EGFR* gene mutations (MS: 58 d, *P*=0.023).

**Conclusion:**

High miR-34 expression in advanced stage NSCLC is the predictive factor for multiple metastatic, adenocarcinoma cell type and adenocarcinoma negative *EGFR* mutation. High expression of miR-155 and miR-222 are poor prognoses, especially high miR-222 found in metastasis M1b and positive *EGFR* mutation and miR-155 found in adenocarcinoma and positive *EGFR* gene mutations. Further studies regarding correlation between miRNA and survival rate are needed.

## Introduction

Lung cancer is the most common cause of cancer related death among men in the world^[1, 2]^. The same thing happened in Indonesia, lung cancer is also a cancer incidence and the cause of death from cancer is mostly in men, while in women it ranks fifth^[2]^. According to data from Dharmais National Cancer Hospital, lung cancer takes the number one cause for cancer related death among men (28.94%). The high mortality of lung cancer is resulted by advance stage as well as resistance of therapy^[3]^. Currently, non-small cell lung cancer (NSCLC) constitutes 85% of all lung cancer cases^[4]^.

Lung cancer remains one of the worst prognosis cancers. One of the causes of high mortality rates is that many lung cancer patients come in advanced stages. Since the very high mortality caused by lung cancer, molecular marker to determine more sensitive therapy among NSCLC patients is needed. In this proteomic era, it is appropriate for more appropriate non-invasive prognostic modalities to be the top priority of the researchers. Identifying new prognostic biological markers is very critical and essential for controlling lung cancer. Moreover, over the past decade, molecular molecules have been found to be non-coding small RNA called micro RNA (miRNA). The miRNA itself decreases the protein expression of the targeted gene by repressing the translation process or degrading messenger RNA (mRNA)^[5]^. In normal cells, miRNA can regulate various processes such as cellular development, differentiation, proliferation, and apoptosis^[6]^. At present more than two thousand types of miRNA have been found in humans that target at least 60% miRNA^[7]^.

In recent years it is known that miRNA can be secreted from within cells and has a role in intercellular transfer through exosomes, apoptotic bodies, and others. MiRNA that circulates in the body through lipid complexes or lipoproteins is very stable in the body^[8]^. This makes the excreted miRNA will have access to body fluids so that the miRNA in circulation can be used as a non-invasive biomarker of cancer^[9]^. MiRNA is not easily degraded and can be found not only in tissues but also in body fluids, including blood (plasma and serum) and sputum^[10]^.

It is undeniable that miRNA plays a role in tumorigenesis. In cancer cells there is a change in miRNA expression which occurs with excessive proliferation and apoptotic resistance^[11]^. So the role of the miRNA in malignancy can be divided into two, namely: oncogenes (oncomiR) or as a tumor suppressor gene. This discovery can make miRNA a new marker in the diagnosis and prognosis of lung cancer^[12]^. This prognosis marker is important to see the possibility of success of a therapy.

MiR-34 is tumor suppressor gene targetting more than 77 RNA messengers (mRNA)^[13]^. It suppresses tumor's growth and metastasis by inhibiting celll cycle process, EMT, metastasis, cancer stem cell and carcinogenesis^[14]^. miR-34 controls MET activity by binding with 3'UTR MET thus MET expression improved^[15]^. miR-34 family also induces p53 excess expression which causes MET expression^[13]^.

MiR-222 expression increases among solid tumors and its oncogenic role is known on NSCLC as well as other cancers. MiR-222 acts as oncomiR that causes anti-EGFR resistence. The higher level of MET expression will promote oncoprotein c-Jun to bind with miR-222, affecting PTEN expression inhibition and TIMP3^[13]^. This inhibition increases migration and invasion as well as resistance towards anti EGFR and chemotherapy^[16]^.

MiR-148b has also been reported to target carcinoembryonic antigen (CEA) mRNA, and regulate NSCLC proliferation and migration through the CEA signaling pathway. Implicitly, the role of miR-148b in regulating CEA expression leads to an argument that miR-148b can also be used as a prognostic parameter for NSCLC as well as the use of CEA^[17]^.

As oncomiR, miR-155 targets tumor suppressor genes, one of which is programmed cell death 4 (*PDCD4*) mRNA^[18]^. PDCD4 suppressor tumor regulates the carcinogenesis process in the form of cell proliferation through the PI3K/Akt pathway and the JAK/STAT pathway. Increased expression of miR-155 on NSCLC will reduce PDCD4 protein expression, as a result there is activation of PI3K/Akt pathway and increased expression of two important proteins in the cascade below, namely CCND1 and CDK4. These two proteins are cell cycle signals in the G_1_/S phase (G_1_/S checkpoint), so that the increase in expression causes an increase in cell proliferation^[19]^.

Reviewing the above explanation, miR-34, miR-148 (tumor suppressor) and miR-155, miR-222 (onkomiR) have a crucial role in the process of carcinogenesis. The results of other studies also state that the expression of the two microRNAs is related to overall survival (OS) in NSCLC patients and has the potential to be used as prognostic parameters. Therefore, in this study we will measure the expression level of these two microRNAs in NSCLC patients in the "Dharmais" Cancer Hospital and are associated with clinical and laboratory status factors. "Dharmais" Cancer Hospital is a national cancer referral center, so it is expected that the samples used can represent the profile of patients throughout Indonesia.

The aim of this study was to find the predictive and prognostic parameters intended to improve therapy management in NSCLC patients.

## Methods

Cohort retrospective was chosen for this study among 52 subjects, which were patients from Dharmais National Cancer Hospital. Sample was obtained from patients' serum. miR-34, miR-148, miR-155 and miR-222 serum are measured through real-time PCR (qPCR). Target gene transcripts are amplified using specific primers. Amplification using a 7500 Fast (Applied Biosystem) machine with the following steps: initial denaturation at 95 ℃ for 10 min, followed by 45 denaturation cycles at 95 ℃ for 10 s and 60 ℃ for 1 min. Melt-curve analysis is performed after 45 qPCR cycles are completed. The amplification results are published in the form of cycle threshold (CT), which is the number of amplification cycles at the time the amplicon is determined to reach the detection threshold (threshold). The level of miRNA expression is expressed as the level of absolute expression and relative expression (fold change). Relative expression is decrement of CT miRNA achieved compared to endogenous control (ΔCT). In this study miR-16 was used as a reference. Fold change is calculated by the formula: 2^-ΔΔCT^ and expressed without units while for the fold regulation value is calculated by calculating the inverse negative of the fold change number previously calculated. So for fold regulation is calculated by the formula fold regulation=-1/(fold change).

Data were analyzed and interpreted with descriptive analysis (presented by tables and graphs), bivariate analysis (using *Mann Whitney-U* for two type of variables or *Kruskal-Wallis* for more than two type of variables). *Kaplan-Meier* analysis was used to know association between patients characteristic which are sociodemographic, clinical and laboratory value and performance status (PS). *Kaplan-Meier* analysis was also used to reveal correlation between miRNA expression and survival rate. This study used significance *P* value of < 0.05 and confidence interval of 95%.

The research conducted has received approval from the medical/health research ethics committee of the "Dharmais" Cancer Hospital with the serial number is 032/KEPK/V/2016.

## Results

Correlation between miR-148 and miR-155 expression with clinicopathological characteristics of advance stage NSCLC patients As shown in Table 1, there were no significant clinicopathological correlations except for PS.

**1:** Correlation between miR-148 and miR-155 expression with clinicopathological characteristics of advance stage NSCLC patients

Characteristic	miR-34			miR-222			miR-148			miR-155	
	Median (Min.: Maks.)	*P*		Median (Min.; Maks.)	*P*		Median (Min.; Maks.)	*P*		Median (Min.; Maks.)	*P*
Gender (*n*=52)	1.125 (0.06; 109.14)	0.968		5.65 (0.04; 5, 789.38)	0.558		15.66 (0.11; 7, 214.42)	0.832		3.66 (0.06; 660.16)	0.912
Male (*n*=37)	1.43 0.06; 09.14)			16.87 (0.04; 12, 914.16)			14.09 (0.11; 3, 508.57)			6.758 (0.06; 426.58)	
Female (*n*=15)	0.97 (0.28; 51.98)			1.94 (0.07; 15, 789.38)			20.63 (0.35; 7, 214.42)			1.307 (0.07; 660.16)	
Age category (*n*=52)	1.13 (0.06; 109.14)	0.344		5.65 (0.04; 15, 789.38)	0.665		15.66 (0.11; 7, 214.42)	0.562		3.657 (0.06; 660.16)	0.685
> 60 years (*n*=20)	1.75 (0.28; 51.98)			17.41 (0.09; 15, 789.38)			17.91 (0.43; 7, 214.42)			7.073 (0.10; 660.16)	
40-60 years (*n*=28)	0.91 (0.06; 109.14)			2.44 (0.04; 9, 519.48)			12.01 (0.35; 3, 251.00)			1.37 (0.06; 145.68)	
< 40 years (*n*=4)	2.36 (0.15; 27.10)			81.74 (0.05; 176.89)			163.19 (0.11; 664.75)			4.531 (0.07; 82.52)	
Smoking (*n*=52)	1.13 (0.06; 109.14)	0.435		5.65 (0.04; 15, 789.38)	0.847		15.66 (0.11; 7, 214.42)	0.457		3.66 (0.06; 660.16)	0.614
Yes (*n*=24)	1.29 (0.06; 44.94)			5.65 (0.05; 250.15)			13.53 (0.11; 883.24)			1.33 (0.07; 219.29)	
No (*n*=28)	1.10 (0.28; 109.14)			8.45 (0.04; 15789.34)			23.65 (0.35; 7, 214.42)			7.07 (0.06; 660.16)	
PS (*n*=52)	1.13 (0.06; 109.14)	0.131		5.65 (0.04; 15, 789.38)	0.018		15.66 (0.11; 7, 214.42)	0.049		3.66 (0.06; 660.16)	0.176
PS 3-4 (*n*=14)	3.46 (0.45; 51.98)			67.60 (0.30; 15, 789.38)			70.39 (0.65; 7, 214.42)			9.99 (0.12; 660.16)	
PS 2 (*n*=19)	0.85 (0.06; 51.27)			1.94 (0.09; 12, 914.16)			12.97 (0.43; 3, 508.57)			1.25 (0.10; 426.58)	
Clinical stage (*n*=52)	1.13 (0.06; 109.14)	0.842		5.65 (0.04; 15, 789.38)	0.652		15.66 (0.11; 7, 214.42)	0.887		3.66 (0.06; 660.16)	0.610
Ⅳb (*n*=28)	0.85 (0.06; 51.27)			3.29 (0.04; 12, 914.16)			17.23 (0.43; 3, 508.57)			1.31 (0.06; 426.58)	
Ⅳa (*n*=21)	1.58 (0.15; 51.98)			36.65 (0.05; 15, 789.38)			23.12 (0.11; 7, 214.42)			14.51 (0.07; 660.16)	
Ⅲb (*n*=3)	1.96 (0.28; 109.14)			0.56 (0.23; 521.55)			4.19 (0.49; 444.69)			0.43 (0.22; 137.88)	
Tumor status (*n*=52)	1.13 (0.06; 109.14)	0.607		5.65 (0.04; 15, 789.38)	0.948		15.66 (0.11; 7, 214.42)	0.570		3.66 (0.06; 660.16)	0.782
T4 (*n*=35)	1.10 (0.06; 51.98)			2.94 (0.04; 15, 789.38)			11.69 (0.11; 7, 214.42)			1.25 (0.06; 660.16)	
T3 (*n*=7)	0.77 (0.20; 109.14)			1.94 (0.07; 521.55)			2.86 (0.35; 444.69)			1.31 (0.07; 137.82)	
T2 (*n*=4)	3.30 (0.73; 9.58			23.39 (0.30; 47.73)			69.37 (1.07; 251.89)			18.53 (0.18; 50.10)	
T1 (*n*=6)	3.48 (0.28; 44.94)			11.94 (0.18; 176.89)			91.31 (0.59; 503.79)			17.26 (0.14; 82.52)	
Nodule status (*n*=52)	1.13 (0.06; 109.14)	0.625		5.65 (0.04; 15, 789.38)	0.742		15.66 (0.11; 7, 214.42)	0.495		3.66 (0.06; 660.16)	0.601
N3 (*n*=13)	0.85 (0.20; 109.14)			2.94 (0.07; 12, 914.16)			11.06 (0.35; 3, 508.57)			1.31 (0.07; 426.58)	
N2 (*n*=36)	1.29 (0.06; 51.98)			5.65 (0.04; 15, 789.38)			16.34 (0.11; 7, 214.42)			3.666 (0.06; 660.16)	
N0 (*n*=3)	2.91 (1.10; 4.56)			67.49 (1.81; 176.89)			325.54 (4.95; 503.79)			51.86 (1.00; 82.52)	
Metastasis status (*n*=52)	1.13 (0.06; 109.14)	0.842		5.65 (0.04; 15, 789.38)	0.652		15.66 (0.11; 7, 214.42)	0.887		3.66 (0.06; 660.16)	0.610
M1b (*n*=29)	0.85 (0.06; 51.27)			3.29 (0.04; 12, 914.16)			17.23 (0.43; 3, 508.57)			1.31 (0.06; 426.58)	
M1a (*n*=20)	1.58 (0.15; 51.98)			36.65 (0.05; 15, 789.38)			23.12 (0.11; 7, 214.42)			14.51 (0.07; 660.16)	
M0 (*n*=3)	1.96 (0.28; 109.14)			0.56 (0.23; 521.55)			4.19 (0.49; 444.69)			0.43 (0.22; 137.82)	
M1b metastatic type (*n*=29)	0.85 (0.06; 51.27)	0.020		3.29 (0.04; 12, 914.16)	0.383		17.23 (0.43; 3, 508.57)	0.074		1.31 (0.06; 426.58)	0.169
Single (*n*=19)	0.77 (0.06; 27.10)			2.94 (0.04; 669.37)			4.95 (0.43; 664.75)			1.25 (0.06; 145.68)	
Multiple (*n*=10)	3.21 (0.59; 51.27)			11.94 (0.30; 12, 914.16)			31.29 (1.07; 3, 508.57)			8.05 (0.18; 426.58)	
M1b metastatic location (*n*=29)	0.85 (0.06; 51.27)	0.176		3.29 (0.04; 12, 914.16)	0.300		17.23 (0.43; 3, 508.57)	0.295		1.31 (0.06; 426.58)	
Brain (*n*=6)	0.70 (0.36; 2.91)			35.39 (0.30; 669.37)			19.61 (0.43; 503.79)			9.55 (0.12; 51.86)	
Liver (*n*=2)	0.81 (0.77; 0.85)			20.25 (0.09; 40.41)			16.63 (0.67; 32.60)			5.59 (0.13; 11.06)	
Bone (*n*=9)	0.66 (0.06; 9.45)			1.43 (0.04; 94.79)			1.97 (0.43; 594.97)			1.00 (0, 06; 145.68)	
Adrenal gland (*n*=2)	13.86 (0.62; 27.10)			96.80 (30.84; 162.77)			333.81 (2.86; 666.75)			8.59 (8.26; 8.92)	
Multiorgan (*n*=10)	3.21 (0.59; 51.27)			11.94 (0.30; 12, 914.16)			31.29 (1.07; 3, 508.57)			8.05 (0.18; 426.58)	
Cancer cell type (*n*=52)	1.13 (0.06; 109.14)	0.009		5.65 (0.04; 15, 789.38)	0.113		15.66 (0.11; 7, 214.42)	0.082		3.66 (0.06; 660.16)	
Adenocarcinoma (*n*=43)	1.50 (0.15; 109.14)			16.87 (0.05; 15, 789.38)			20.63 (0.11; 7, 214.42)			8.26 (0.07; 660.16)	
Squamous cell carcinoma (*n*=9)	0.66 (0.06; 2.16)			0.89 (0.04; 217.77)			1.97 (0.53; 190.90)			0.20 (0.06; 89.06)	
Adenocarcinoma: *EGFR* mutation status (*n*=43)	1.50 (0.15; 109.14)	0.031		16.87 (0.05; 15, 789.38)	0.064		20.63 (0.11; 7, 214.42)	0.168		8.26 (0.07; 660.16)	
Positive (*n*=10)	0.81 (0.15; 4.56)			1.67 (0.05; 176.89)			8.57 (0.11; 503.79)			0.79 (0.07; 82.52)	
Negative/Wild type (*n*=33)	2.35 (0.28; 109.14)			25.58 (0.07; 15, 789.38)			33.28 (0.35; 7214.42)			8.20 (0.07; 660.16)	
*EGFR* mutation status (*n*=52)	1.125 (0.06; 109.14)	0.153		5.65 (0.04; 15, 789.38)	0.18		15.66 (0.11; 7214.42)	0.335		3.66 (0, 06; 660, 16)	
Positive (*n*=10)	0.81 (0.15; 4.56)			1.67 (0.05; 176.89)			8.57 (0.11; 503.79)			0.79 (0.07; 82.52)	
Negative/Wild type (*n*=42)	1.58 (0.06; 109.14)			15.24 (0.04; 15, 789.38)			17.91 (0.35; 7214.42)			7.51 (0.06; 660.16)	
Comparison was determined by *Wilcoxon* rank sum test or *Kruskal-Walls H* rank sum test. *P* value < 0.05 was considered as statistically significant. PS: performance status; epidermal growth factor receptor.											

Association of serum miR-34, miR-222, miR-148 and miR-155 with overall survival. To evaluate prognostic roles of serum miR-34, miR-222, miR-148 and miR-155, we collected survival records of 52 patients with median follow-up 12 months, range (1 to 12 months). We divided all patients into patients with miR-34, miR-222, miR-148 or miR-155 high expression and patients with low expression subgroups based on their median value. 46.15% (24 out of 52 patients) had miR-34 expressions higher than median value, whereas 32.69% (17 out of 52 patients) had miR-222 expressions higher than median value. 23.08% (12 out of 52 patients) had miR-148 expressions higher than median value, whereas 40.38 % (21 out of 52 patients) had miR-155 expressions higher than median value (Fig 1-6 and Tab 1-7).

**1:**
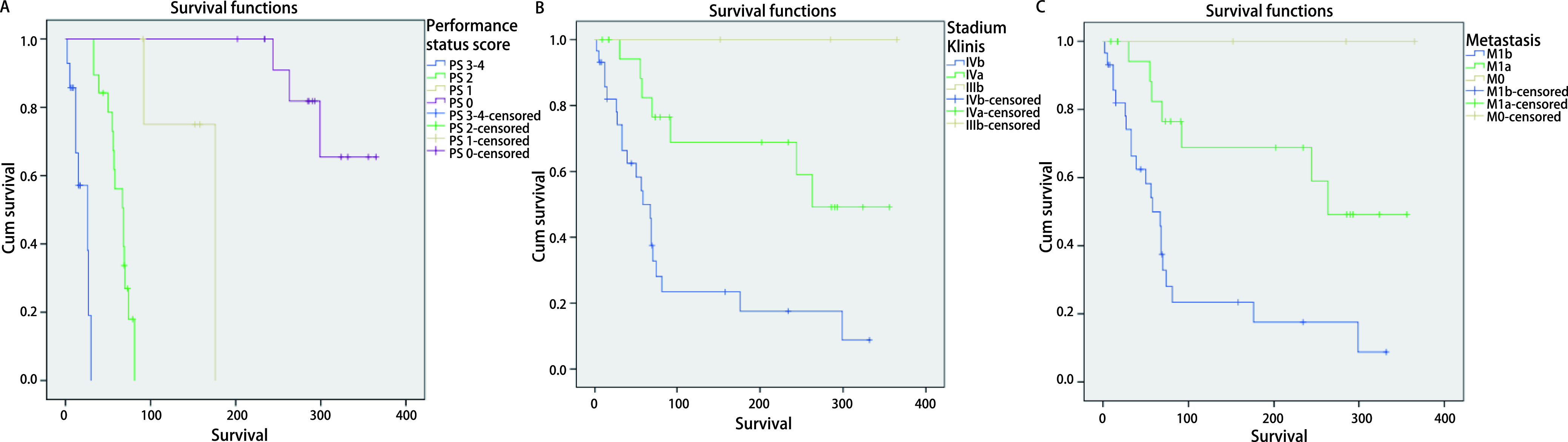
*Kaplan-Meier* analysis of the factors affecting OS in advanced stage NSCLC patients. A: PS (*P* < 0.000, 01). Median survival: PS 3-4: 26 d; PS 2: 68 d; PS 1: 176 d; PS 0: > 365 d. 1 year survival rate: PS 3-4: 42.9%; PS 2: 21.1%; PS 1: 60%; PS 0: 78.6%. B: clinical stage (*P*=0.000, 78). Median Survival: IVb: 58 d; IVa: 263 d; IIIb: > 365 d; 1 year survival rate: IVb: 27.6%; IVa: 65%; IIIb: 100%; C: Metastasis (*P*=0.000, 78). Median survival: M1b: 58 d; M1a: 263 d; M0: > 365 d; 1 year survival rate: M1b: 27.6%; M1a: 65%; M0: 100%. OS: overall survival; PS: performance status; NSCLC: non-small cell lung cancer.

**2:**
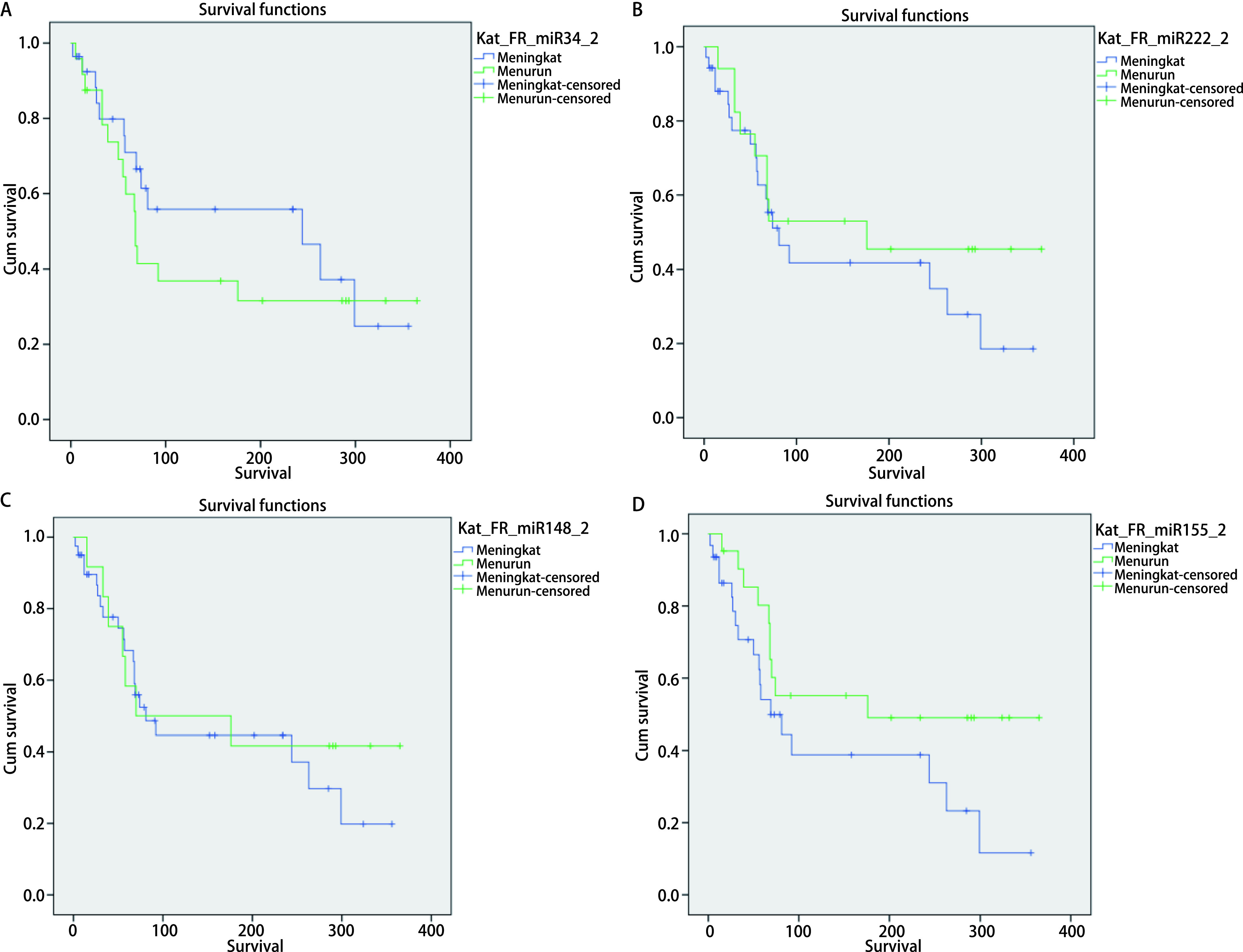
Analysis of *Kaplan-Meier's* survival for miR-34, miR-222, miR-148 and miR-155 serum expression variable (fold regulation) in advanced stage NSCLC. A: Survival curve of serum miR-34 levels (fold regulation) (*Log-rank* test, *P*=0.416). Median survival: High (equal or more higher than median): 244 d; Low (less than median): 68 d; 1 year survival rate: High (equal or more higher than median): 53.6%; Low (less than median): 37.5%. B: Survival curve of serum miR-222 levels (fold regulation)(*Log-rank* test, *P*=0.367). Median survival: High (equal or more higher than median): 81 d; Low (less than median): 176 d; 1 year survival rate: High (equal or more higher than median): 45.7%; Low (less than median): 47.1%. C: Survival curve of serum miR-148 levels (fold regulation) (*Log-rank* test, *P*=0.614): Median survival: High (equal or more higher than median): 81 d; Low (less than median): 70 d; 1 year survival rate: High (equal or more higher than median): 47.5%; Low (less than median): 41.7%. D: Survival curve of serum miR-155 levels (fold regulation)(*Log-rank* test, *P*=0.099). Median survival: High (equal or more higher than median): 69 d; Low (less than median): 176 d; 1 year survival rate: High (equal or more higher than median): 41.9%; Low (less than median): 52.4%.

**3:**
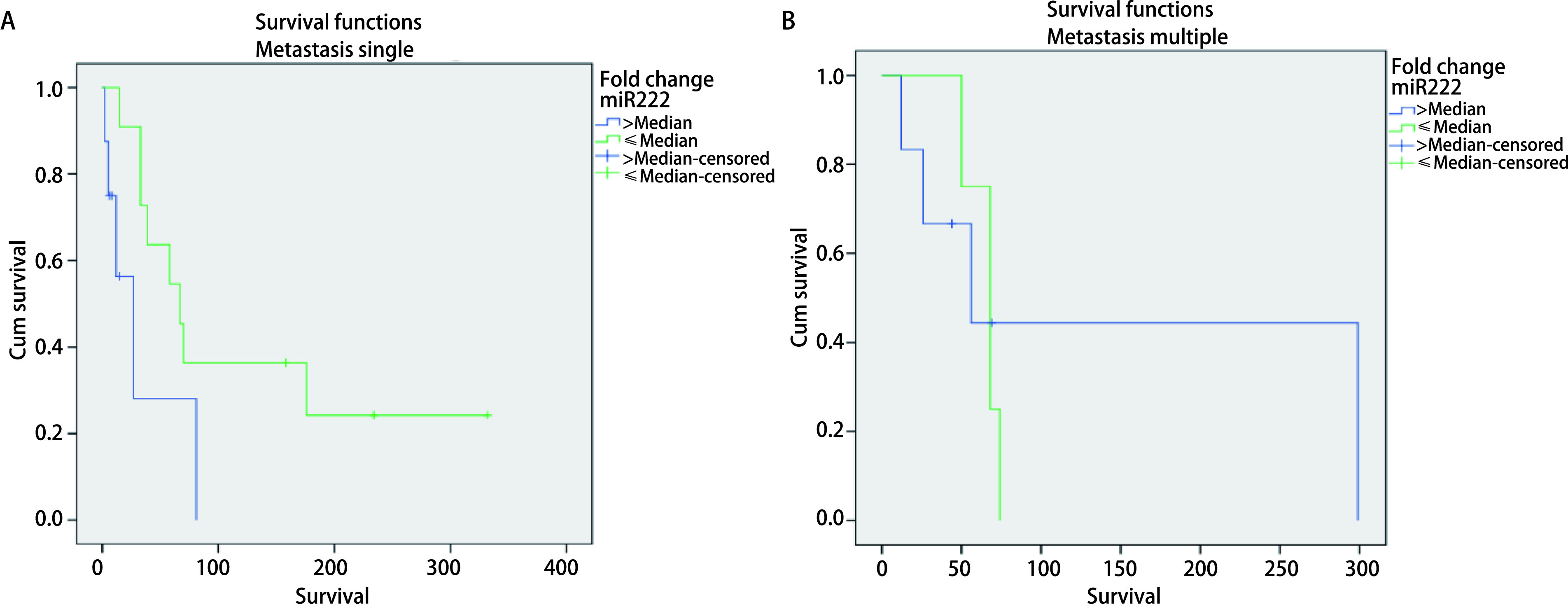
Analysis of *Kaplan-Meier's* survival for miR-222 serum expression variable (fold change) in advanced stage NSCLC with metastasis M1b. A: metastasis single (*Log-rank* test, *P*=0.049). Median survival: High (equal or more higher than median): 27 d; Low (less than median): 67 d. 1 year survival rate: High (equal or more higher than median): 37.5%; Low (less than median): 27.3%. B: Metastasis multiple (*Log-rank* test, *P*=0.761): Median survival: High (equal or more higher than median): 56 d; Low (less than median): 68 d. 1 year survival rate: High (equal or more higher than median): 33.3%; Low (less than median): 0%.

**4:**
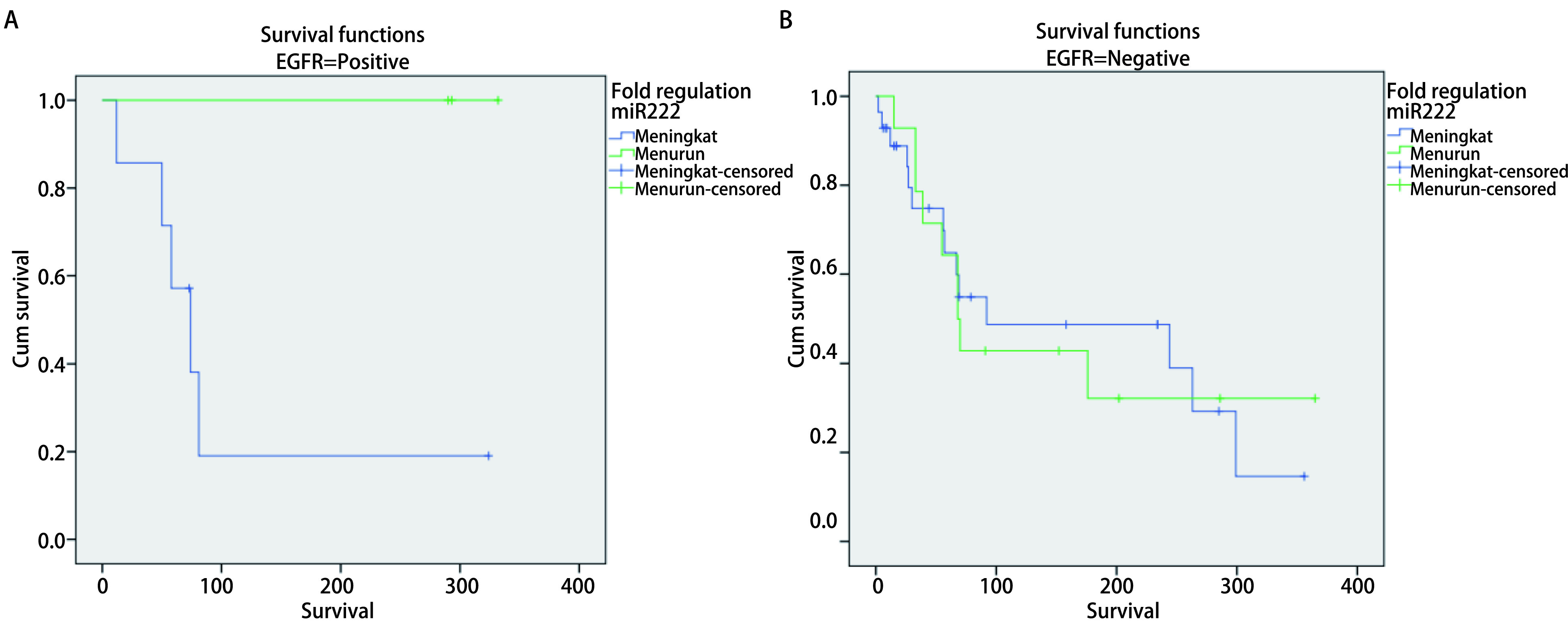
Analysis of *Kaplan-Meier's* survival for miR-222 serum expression variable (fold regulation) in advanced stage NSCLC with positive *EGFR* gene mutation. A: EGFR positive (*Log-rank* test, *P*=0.049, 95). Median survival: High (equal or more higher than median): 74 d; Low (less than median): 293 d; 1 year survival rate: High (equal or more higher than median): 28.6%; Low (less than median): 100.0%. B: EGFR negative (*Log-rank* test, *P*=0.962). Median survival: High (equal or more higher than median): 92 d; Low (less than median): 68 d; 1 year survival rate: High (equal or more higher than median): 50.0%; Low (less than median): 35.7%.

**5:**
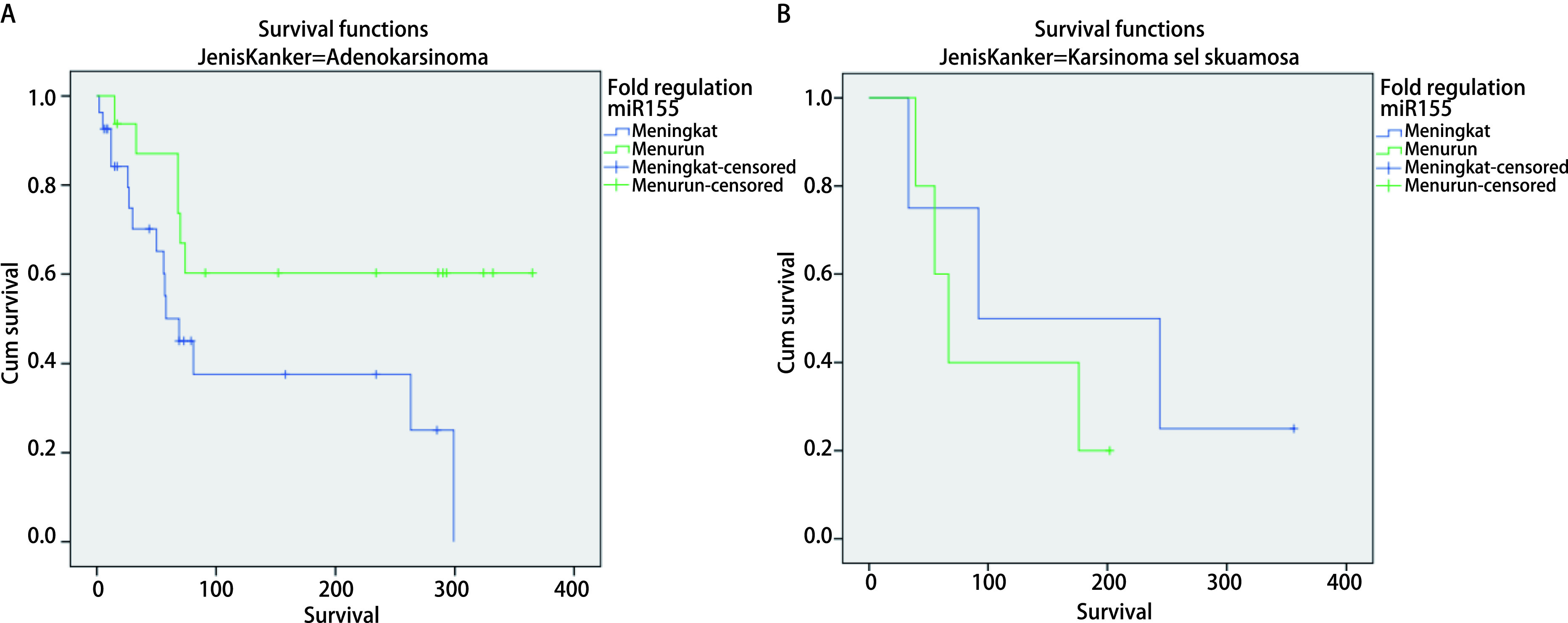
Analysis of *Kaplan-Meier's* survival for miR-155 serum expression variable (fold regulation) in advanced stage NSCLC with adenocarcinoma. A: Adenocarcinoma (*Log-rank* test, *P*=0.034). Median survival: High (equal or more higher than median): 69 d; Low (less than median): > 365 d. 1 year survival rate: High (equal or more higher than median): 44.4%; Low (less than median): 62.5%. B: Squamous cell carcinoma (*Log-rank* test, *P*=0.484). Median survival: High (equal or more higher than median): 92 d; Low (less than median): 67 d. 1 year survival rate: High (equal or more higher than median): 25.0%; Low (less than median): 20.0%.

**6:**
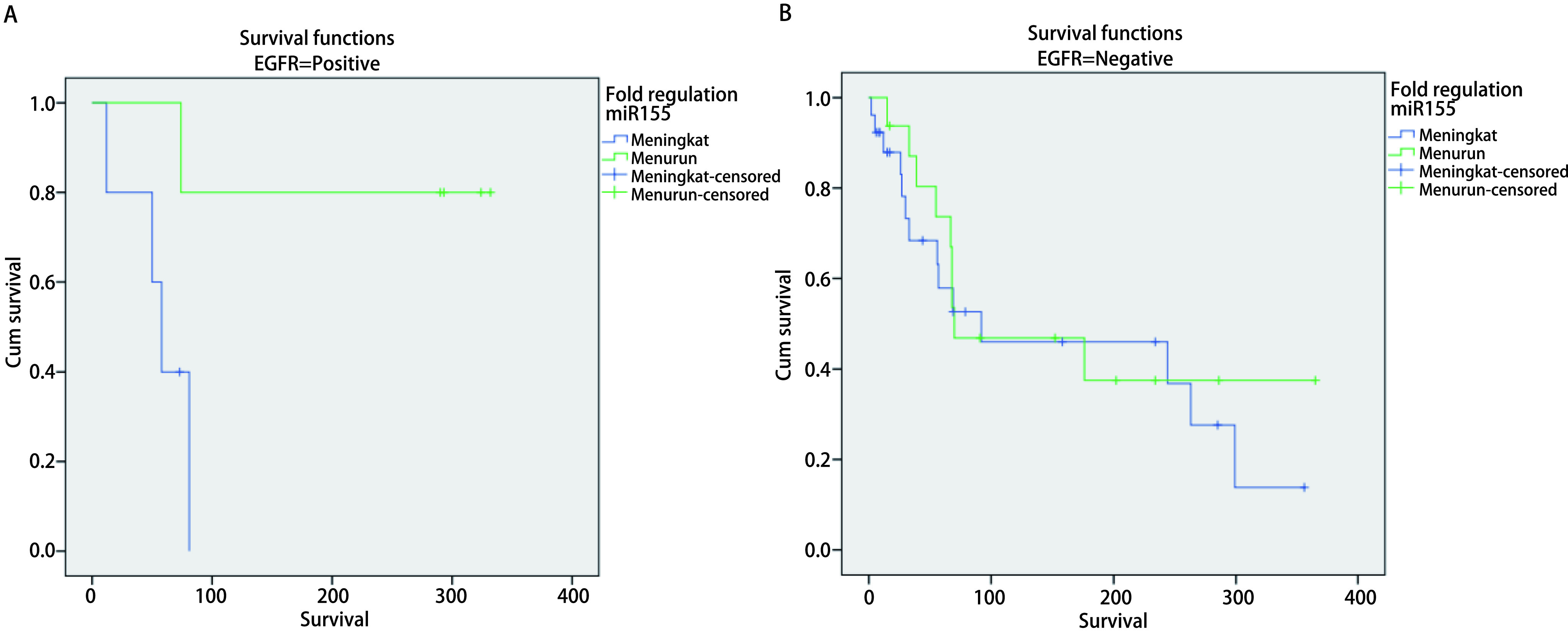
*Kaplan-Meier's* survival analysis curve for miR-155 serum expression characteristic (fold regulation) in advanced stage NSCLC with positive EGFR gene mutation. A: EGFR positive (*Log-rank* test, *P*=0.023). Median survival: High (equal or more higher than median): 58 d; Low (less than median): 332 d; 1 year survival rate: High (equal or more higher than median): 20.0%; Low (less than median): 80.0%. B: EGFR positive (*Log-rank* test, *P*=0.657). Median survival: High (equal or more higher than median): 92 d; Low (less than median): 70 d; 1 year survival rate: High (equal or more higher than median): 46.2%; Low (less than median): 43.8%.

**2:** *Cox* regression analysis of the factors affecting OS in advanced stage NSCLC patients

Clinicopathology characteristics	Hazard ratio (HR)	HR (95%CI)		*P*	
Gender					
Male	0.83	0.39-1.76		0.626	
Female	1.00				
Age group					
< 40 years	1.00	0.98-1.04		0.59	
40-60 years	1.01				
> 60 years	1.01				
Smoking					
Yes	1.73	0.91-3.31		0.095	
No	1.00				
PS					
PS 3-4	3.65	-		< 0.001	
PS 2	2.55	1.19-5.46			
PS 1	0.76	0.26-2.17			
PS 0	1.00	0.02-0.23			
Clinical stage					
IVb	2.99		1.63-5.27		0.0001
IVa	2.99				
IIIb	1.00				
Tumor (T)					
T4	1.23		0.89-1.71		0.184
T3	1.23				
T2	1.23				
T1	1.00				
Nodule (N)					
N3	1.15		0.63-2.11		0.655
N2	1.15				
N0	1.00				
Metastasis (M)					
M1b	2.96		1.58-5.52		0.0002
M1a	2.96				
M0	1.00				
Cancer cell type					
Adenocarcinoma	1.00		0.38-1.94		0.70
Squamous cell carcinoma	0.86				
*EGFR* mutation status					
Positive	1.00		0.16-1.10		0.054
Negative	0.42				
M1b metastatic type					
Single	1.00		0.38-2.00		0.74
Multiple	0.88				

**3:** *Cox* regression's survival analysis for miR-34, miR-222, miR-148 and miR-155 serum expression characteristic (fold regulation) in advanced stage NSCLC

Characteristic of miRNA	Hazard ratio (HR)	HR (95%CI)	*P*
Serum miR-34 levels			
High (equal or more higher than median)	1.00	(0.54-1.96)	0.416
Low (less than median)	1.03		
Serum miR-222 levels			
High (equal or more higher than median)	0.72	(0.38-1.37)	0.367
Low (less than median)	1.00		
Serum miR-148 levels			
High (equal or more higher than median)	1.00	0.32-1.16	0.613
Low (less than median)	0.61		
Serum miR-155 levels			
High (equal or more higher than median)	0.75	0.39-1.42	0.099
Low (less than median)	1.00		

**4:** Analysis of *Cox* regression's survival for miR-222 serum expression variable (fold change) in advanced stage NSCLC with metastasis M1b

Variable	Hazard ratio (HR)	HR (95%CI)	*P*
Serum miR-222 levels (*n*=19)			
High (equal or more higher than median) (*n*=8)	0.44	(0.37-0.97)	0.049*
Low (less than median) (*n*=11)	1.00		

**5:** Analysis of *Cox* regression's survival for miR-222 serum expression variable (fold regulation) in advanced stage NSCLC with positive *EGFR* gene mutation

Variable	Hazard Ratio (HR)	HR (95%CI)	*P*
Serum miR-222 levels (*n*=10)			
High (equal or more higher than median) (*n*=7)	0.73	(0.54-0.98)	0.049*
Low (less than median) (*n*=3)	1.00		

**6:** *Cox* regression's survival analysis for miR-155 serum expression characteristic (fold regulation) in advanced stage NSCLC with adenocarcinoma

Characteristic of miRNA	Hazard ratio (HR)	HR (95%CI)	*P*
Serum miR-155 levels (*n*=43)			
High (equal or more higher than median) (*n*=27)	0.35	0.30-0.89	0.034*
Low (less than median) (*n*=16)	1.00		

**7:** *Cox* regression's survival analysis for miR-155 serum expression characteristic (fold regulation) in advanced stage NSCLC with positive *EGFR* gene mutation

Characteristic of miRNA	Hazard Ratio (HR)	HR (95%CI)	*P*
Serum miR-155 levels (*n*=10)			
High (equal or more higher than median) (*n*=5)	0.73	0.27-0.84	0.023*
Low (less than median) (*n*=5)	1.00		

## Discussion

In this study, the expression of miRNA to be examined is sufficient to only be taken from blood serum because it is relatively easy to obtain with non-invasive procedures.

The most advanced NSCLC stage obtained T4N2M1b stage IVb multiorgan metastasis with positive *EGFR* gene mutation (19.2%) and exon 19 (9.6%). The increasing clinical stage of the prognosis is poor, this classification of stages is very useful for determining prognosis and therapeutic strategies^[20, 21]^. *EGFR* gene mutations were 19.2% and most were exon 19 and exon 21. *EGFR* gene mutations were associated with several characteristics such as mutations more frequently in female patients, adenocarcinoma type NSCLC, non-smokers/those who had quit smoking and East Asian patients^[22, 23]^. 85%-90% of *EGFR* mutations are exon 19 deletions and L858R mutations in exon 21^[24]^. Excessive activity of EGFR signals is associated with poor progression and prognosis in NSCLC.

This study found high miR-34 expression serum (46.15%), high miR-222 expression (32.69%), miR-148 expression (23.08%) and miR-155 expression (40.38%). The high miR-222 expression serum was related statistically significantly to clinical deterioration of PS (*P*=0.018). Mir 222/221 is a regulator of the proliferation of NSCLC cell development through the P57 target. MIR-222, 221 inhibition is a potential therapy in NSCLC^[25]^. Increased expression of miR 222 in NSCLC indicates the presence of prognostic factors and poor survival. The expression level of miR 222 is useful as a biomarker in the use of target therapy and cases that require special attention to NSCLC^[26]^. This study found serum miRNA, namely miR-148 with high expression (23.08%), low expression (76.92%) and miR-155 with high expression (40.38%), low expression (59.62%). The low expression of miR-148 was associated with poor overall survival (OS) in 151 NSCLC patients so miR-148 could be used as a NSCLC patient prognostic factor^[27]^. The high expression of miR-155 was said to be associated with a poor prognosis in 317 NSCLC patients in Maryland ^[28]^.

Serum miRNA expression on pathological diagnosis variables was statistically significant with an increase in the expression of miR-34 as a predictive biomarker for the type of metastasis M1b (*P*=0.020), adenocarcinoma cancer cell types (*P*=0.009) and adenocarcinoma negative *EGFR* mutation (*P*=0.031). Another study found that miR-34b caused hypermethylation in 41% of NSCLC specimens, multivariate methylation analysis of miR-34b was significantly associated with distant metastasis of lymphatic invasion^[29]^. Most miRNA expressions increase in adenocarcinoma cell types and the 5 largest genes are miR-181a, mir-191, miR-107, miR-103 and let-7b^[30]^.

Serum miRNA with clinical characteristics found statistically significant results in high miR-148 expression on characteristics of PS (*P* < 0.001). The high expression of miR-148 serum can cause clinical deterioration in NSCLC patients so that the characteristics of performance status are used as the second prognostic factor most often used and measured on the Karnofsky scale^[31]^. Poor performance status is caused by cancer patients who have cachexia which causes a reduced tolerance to cancer treatment and reduced quality and length of life^[32]^.

Although the miR-34, miR-222, miR-148 and miR-155 serum expression were not statistically significant associated with survival rate (miR-34, *P*=0.416; miR-222, *P*=0.367, miR-148, *P*=0.613; miR-155, *P*=0.099), there were theoritically relevant, in which low expression of miR-34 and miR-148 were associated with better outcome of survival rate, while high expression of miR-222 and miR-155 were associated with worse outcome of survival rate among NSCLC patients.

In this study more focused on clinicopathological variables that were found to be significant in relation to miR-222 both in fold change and fold regulation and. It is a biomarker of poor prognosis in cases of metastasis M1b (*P*=0.048, 87) with high expression of miR-222 (MS 27 d, 1 year SR 37.5%), low expression of miR-222 (MS 67 d, 1 year SR 27.5%) and in the case of a positive *EGFR* mutation (*P*=0.049, 95) with high expession of miR-222 (MS 74 d, 1 year SR 28.6%), low expression of miR-222 (MS 293 d, 1 year SR 100.0%). Other studies have found that miR-222 expression is increased in various solid tumors and their oncogenic role is known in NSCLC, cells that express excess miR-222 are TRAIL-resistant and show increased migration and invasion ability^[33]^. Increased expression of miR-222 in NSCLC indicates the presence of prognostic factors and poor survival^[26]^. OncomiR miR-222/221 which causes anti-EGFR resistance and chemotherapy^[13]^. The expression level of miR 222 is useful as a biomarker in the therapeutic use of the target NSCLC^[16]^.

High expression of miR-155 which was found to be statistically significant as a biomarker for poor prognosis was in adenocarcinoma (*P*=0.034) with a high expression of miR-155 (MS 69 d, 1 year SR 44.4%) compared to low expression of miR-155 (MS 365 d, 1 year SR 62.5%) and positive *EGFR* gene mutations (*P*=0.023) with high expression of miR-155 (MS 58 d, 1 year SR 20.0%) compared to low expression of miR-155 (MS 332 d, 1 year SR 80.0%).

Thus, further studies are necessarily needed on serial examination of molecular biology of miRNA expression, not only miR-34, miR-222, miR-148 and miR-155.

## Conclusion

MiRNA is not only in tissue but also in body fluids, including serum.

In the advanced stage NSCLC found the high miR-222 and miR-148 serum expression can cause clinical deterioration or poor Performance Status in patients.

Level of high miR-34 and miR-148 expression associated with better outcome of survival rate, while high expression of miR-222 and miR-155 associated with worse outcome of survival rate among NSCLC patients.

The high expression of miR-34 was found as predictive marker in M1b metastasis, adenocarcinoma cell type and adenocarcinoma negative *EGFR* mutation in the advanced stage NSCLC.

The low expression of miR-34, miR-148 and high expression of miR-222, miR-155 are poor prognoses. The high expression of miR-222 and miR-155 was found as poor prognosis especially in M1b metastasis, adenocarcinoma cell type and positive *EGFR* gene mutation cases.

This study revealed that performance status score, cancer cell type, clinical stage, as well as metastasis status can be predictors for survival rate among NSCLC patients. Further studies regarding correlation between miRNA and survival rate are needed.
